# Multidrug-Resistant Musculoskeletal Tuberculosis: An Aggressive Clinical, Radiological and Molecular Confirmation With Genotypic Drug Susceptibility Testing Approach

**DOI:** 10.7759/cureus.28720

**Published:** 2022-09-03

**Authors:** Vikram Sethi, Shailendra Raghuvanshi, Aarti Kotwal, Rakhee Sodhi Khanduri, Varuna Jethani

**Affiliations:** 1 Microbiology, Himalayan Institute of Medical Sciences, Swami Rama Himalayan University, Dehradun, IND; 2 Radiodiagnosis, Himalayan Institute of Medical Sciences, Swami Rama Himalayan University, Dehradun, IND; 3 Respiratory Medicine, Himalayan Institute of Medical Sciences, Swami Rama Himalayan University, Dehradun, IND

**Keywords:** musculoskeletal tuberculosis, lpa, multidrug resistant, nontuberculous mycobacteria, mycobacterium tuberculosis

## Abstract

Introduction

Increasing evidence suggests that musculoskeletal tuberculosis (MSTB) causes significant morbidity due to the late presentation of symptoms and lack of accurate diagnosis. We aimed to assess the utility of two modalities, viz. radiology and molecular methods, in the early diagnosis of MSTB. Also, the rate of resistance to two basic first-line antitubercular drugs in musculoskeletal TB cases among clinically suspected patients was analyzed.

Methods

Samples from 119 patients with clinical suspicion of musculoskeletal TB were included. A radiological workup of patient and smear microscopy, mycobacterial culture, real-time multiplex polymerase chain reaction (PCR), cartridge based nucleic acid amplification test (CBNAAT), and line probe assay (LPA) of samples were carried out.

Results

Maximum positivity (69.74%) was observed by real-time multiplex, followed by CBNAAT and LPA (68.9%), mycobacterial culture (40.3%), and smear microscopy (19.3%). One additional advantage of using multiplex PCR was the detection of non-tuberculous mycobacteria (NTM) isolate. Forty-five strains (54.9%) on LPA were susceptible to rifampicin and isoniazid, eight (9.8%) were rifampicin mono-resistant, seven (8.5%) were isoniazid (INH) mono-resistant, and 22 (26.8%) were multidrug resistant.

Conclusions

MSTB diagnosis can be expedited by the combination of radiology and molecular methods. The positivity rate escalates, turnaround time improves, and the additional advantage of detection of drug resistance is added when this algorithm is included for clinching the diagnosis of MSTB.

## Introduction

Tuberculosis, caused by *Mycobacterium tuberculosis* (MTB) is primarily notorious for causing pulmonary tuberculosis (PTB), whereas extrapulmonary tuberculosis (EPTB) along with its impact on other sites of the body such as the brain, bones, spine, and lymph nodes, are still lesser explored areas of research. Musculoskeletal TB (MSTB), which comprises around 2% of all TB cases and 11% of extrapulmonary cases, is one of the most common sites involved after PTB. Lack of appropriate diagnostic guidelines makes diagnosing MSTB, both spinal and extra-spinal, a burdensome task for the clinicians [1]. Musculoskeletal sites are usually affected by *Mycobacterium tuberculosis* either from primary sites like lung or from other extrapulmonary sites like lymph nodes, kidney, etc. Exclusive extrapulmonary musculoskeletal TB is rarely reported and often missed. The reasons are low clinical suspicion, indolent nature of the disease, late presentation, nonspecific signs and symptoms initially, paucibacillary nature, inaccessible sites, nonspecific laboratory markers used for diagnosis, etc. [2,3].

Drug resistance, as in PTB, is globally a major concern with MTB too. India, with a large pool of mono-resistant along with rising multidrug-resistant TB (MDR-TB) patients, needs an active approach to tackle the problem. In 2015, India reported 20,763 patients with MDR-TB [4]. The rise was exponential in 2016 with 3.9% of new and 21% of previously treated cases being MDR. The alarming was that 9.5% of these cases eventually had extensively drug-resistant TB, which meant that MDR-TB patients now started having resistance to fluoroquinolone and at least one of the three second-line anti-tuberculosis injectable drugs, (capreomycin, kanamycin or amikacin). However, data on EPTB was still limited due to unavailability of drug susceptibility testing (DST) results from majority of states. Lack of facilities and expertise for image-guided sampling as well as the non-availability molecular methods at most centres further compounded the diagnostic challenge posed by EPTB [5].

There is dearth of studies, to the best of our knowledge on correlation of clinical suspicion and radiological screening followed by microbiological confirmation in MSTB and studies on drug resistance in MSTB has hardly ever been reported. This indicates paucity of knowledge and awareness about this spectrum of TB. Hence, it is imperative that instead of empirically starting an anti-tubercular regiment, a confirmatory diagnosis by following an algorithm of clinico-radiological correlation, and microbiological validation is done.

## Materials and methods

An observational cross-sectional study was conducted at a tertiary care center in North India from August 2020 to August 2021 after ethical clearance (Research Committee, Himalayan Institute of Medical Sciences, SRHU/HIMS/RC/2021/264). Hundred and nineteen samples from different sites viz. spine, knee, wrist, hand, ankle/foot, ileum, pubis, rib, femur, shoulder, sacroiliac joint, and soft tissue/muscle from patients with clinical and radiological suspicion of MSTB were obtained under radiological guidance. All those patients who were on antitubercular treatment (ATT) in the past year or had a history of ATT in the past fifteen days or radiologically confirmed cases of pulmonary tuberculosis were excluded from the study. All samples were digested and decontaminated before processing.

Smear microscopy and mycobacterial culture

Samples were stained using the auramine staining technique and examined by fluorescent microscopy. Samples were subjected to liquid culture (BD Diagnostics, Franklin Lakes, USA) and solid culture media.

Real-time multiplex polymerase chain reaction (PCR)

PCR was performed using a multiplex PCR commercial kit (TRUPCR® MTBC One Step Nested Kit, 3B Black Bio Biotech, India Ltd; MTB/NTM real-time PCR detection). A master mix was prepared using 14 µl multiplex master mix, 3 µl of *Mycobacterium tuberculosis* complex (MTC) primer probe mix, 3 µl of NTM primer probe mix, and 1.5 µl of internal control prime probe mix. Then 21.5 µl of the master mix was transferred into PCR tubes, and 8.5 µl of extracted DNA was added. For the positive control, 21 µl PCR master mix, 1 µl of positive control, and 7.5 µl of nuclease-free water were added to make up the final volume of 30 µl. A thermal cycling profile of one cycle at 37°C for five minutes followed by one cycle at 94°C for 10 minutes, 10 cycles at 94°C for 30 seconds, at 68°C for 30 seconds, at 72°C for 45 seconds, and finally 30 cycles at 94°C for 30 seconds and at 59°C for 60 seconds was used.

Cartridge based nucleic acid amplification test (CBNAAT) and line probe assay (LPA)

GeneXpert® *Mycobacterium tuberculosis*/rifampicin (MTB/RIF) or Truenat® assay was performed as per the manufacturer's instructions [6,7]. 

LPA test was performed using GenoType® MTBDRplus Ver 2 (Hain Lifescience, Nehren, Germany) according to the manufacturer's instructions. To begin with, 500 µL of decontaminated and digested specimen were centrifuged at 10000×g for 15 min, pellet resuspended in 100 µl of lysis buffer, and incubated in a dry bath at 95°C for five minutes. Then, 100 µl of neutralizing buffer was added to the tube and vortexed, centrifuged for five minutes and 5 µl of DNA was used for PCR followed by reverse hybridization. Fifteen minutes of denaturation at 95°C, followed by 10 cycles of denaturation at 95°C for 30 seconds and annealing and extension at 65°C for two minutes were performed, followed by 20 cycles of denaturation at 95°C for 25 seconds, annealing at 50°C for 40 seconds and extension at 70°C for 40 seconds. Amplicons were subjected to reverse hybridization with probe-coated strips in a twincubator. Strips were washed and treated with streptavidin-conjugated alkaline phosphatase; strips were removed with tweezers and allowed to air dry. The strips were analyzed and interpreted by comparing them with the template sheet provided in the kit. The absence of any wild-type band with or without the presence of a mutated (MUT) band was taken to indicate a resistant strain [8].

Statistical analysis

Data were analyzed using Excel software (Microsoft® Corp., Redmond, USA). Sensitivity, Specificity, negative predictive value, and positive predictive values were calculated using a 2×2 table.

## Results

All consecutive clinically suspected MSTB suspects from various out-patient departments and hospital wards between August 2020 till August 2021 were enrolled and subjected to radiological musculoskeletal imaging. A written/informed consent was obtained from the patients and only those enrolled in the study who had the symptoms and radiological signs of MSTB on plain anteroposterior (AP)/lateral radiographs of the involved site, viz. vertebral, knee, hip, wrist, ankle, ileum, rib, pubis, femur, sacroiliac joint and soft tissue/muscle involvement or in case of uncertainty, contrast-enhanced MRI was available. Diagnostic imaging of the chest X-ray/high-resolution computed tomography (HRCT) was done to rule out PTB.

Among 119 patients, 83 (70%) had radiological findings suggestive of MSTB, the most common being joint effusion (47%), soft tissue swelling (33.3%), bony erosions (8.6%), vertebral collapse (6.1%), synovitis (3.7%) and paravertebral abscess (1.2%). Thirty-six (30%) samples with clinical suspicion of MSTB had no findings indicative of TB on imaging. All samples were subjected to fluorescent staining for MTB, culture, real-time multiplex PCR, CBNAAT, and LPA. Among 119 samples, 83 image-guided and 36 blind biopsies were collected. The maximum number of samples were from the spinal region, others being bone and joints, psoas abscess, synovial fluid, etc. All samples were representative of the suggestive clinical findings.

**Figure 1:**
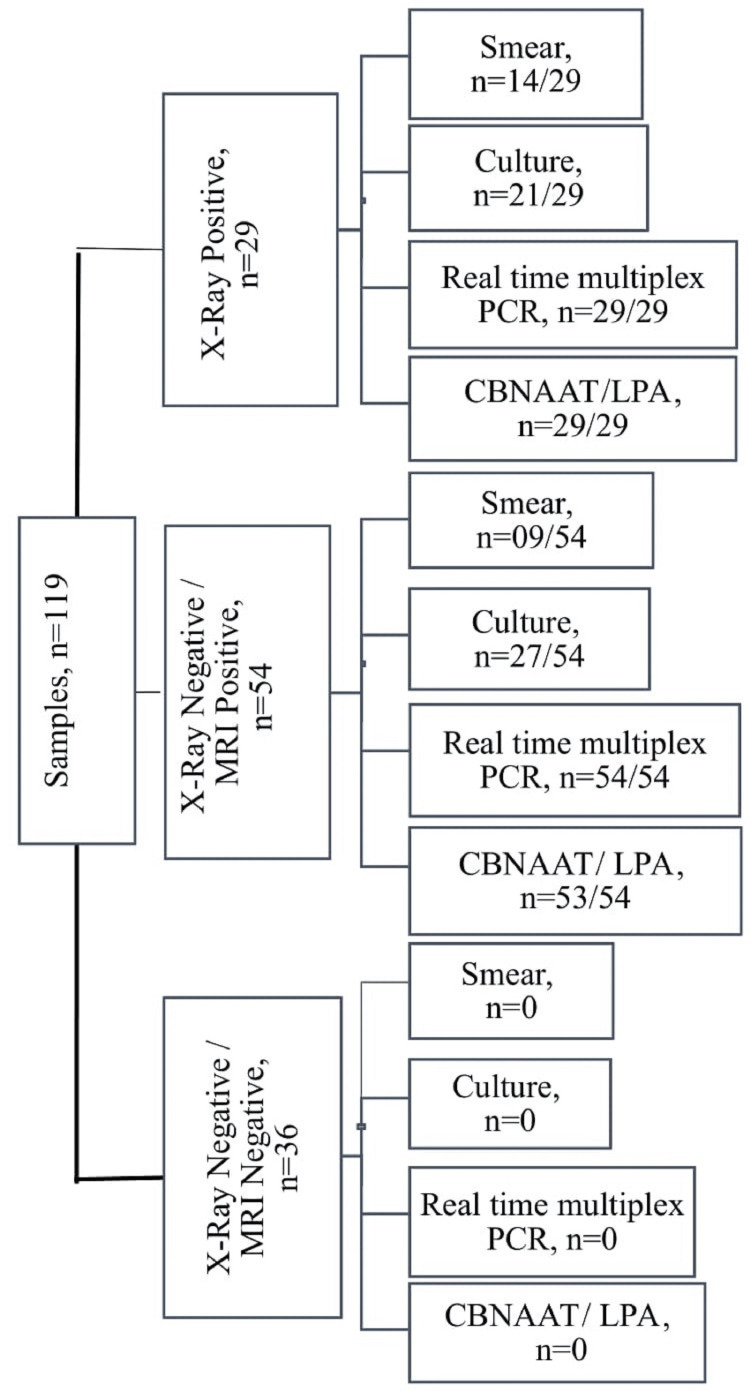
X-ray, MRI, smear microscopy, culture, real-time multiplex PCR, CBNAAT and LPA results PCR - polymerase chain reaction; CBNAAT - cartridge based nucleic acid amplification test; LPA - line probe assay; NTM - nontuberculous mycobacteria; MTB: *Mycobacterium tuberculosis*

The most common site affected was the lumbar vertebra, followed by cervical, bone, chest, and abdominal sites, respectively. The most common symptoms noted were pain (98%), followed by swelling (85%), and restricted movement (67%). Among 119 samples, 19.32% were smear-positive, whereas 40.33% of samples grew *Mycobacterium tuberculosis* on culture. The positivity rate increased markedly by using molecular methods, e.g., real-time multiplex PCR, CBNAAT, and LPA, respectively. All smear-positive samples (n=23, 19.32%) showed 100% concordance, with all the smear-positives being culture positives, too, with none of the smear-positive samples being culture-negative. Also, all smear-positive samples had 100% concordance with real-time multiplex PCR, CBNAAT, and LPA, with all smear-positive samples being positive by all molecular methods except one sample being smear-positive, culture-positive but negative on CBNAAT and LPA. The sample amplified NTM on teal-time multiplex PCR (Figure 1).

Among 119 samples, 25 (21.0%) smear-negative samples were culture-positive. These 25 smear negative-culture positive samples were real-time multiplex PCR, CBNAAT, and LPA positive too. Likewise, out of 71 (59.7%) smear-negative culture-negative samples, 35 samples (49.3%) were positive by real-time multiplex PCR and CBNAAT and showed positive MTB bands on LPA too. Amongst all the samples that tested positive by different modalities, the maximum positivity was observed in samples collected from spinal sites. Out of 48 samples collected from spinal sites, 85.4% and 2.1% of samples were positive for MTB and NTM, respectively. The sensitivity and specificity of various modalities as compared to a gold standard in our study were 48% and 100% for smear microscopy, 100% and 50.7% for real-time multiplex PCR, 98% and 50.7% for CBNAAT, and 98% and 50.7% for LPA, respectively (Table 1).

**Table 1:** Comparison of sensitivity, specificity, PPV and NPV of smear microscopy, real-time multiplex PCR, CBNAAT and LPA while culture considered to be a gold standard PCR - polymerase chain reaction; CBNAAT - cartridge based nucleic acid amplification test; LPA - line probe assay; NTM - nontuberculous mycobacteria; PPV - positive predictive value; NPV - negative predictive value

Culture, n=119	Smear microscopy, n=119		Real-time multiplex PCR, n=119		CBNAAT, n=119		LPA, n=119	
	Positive	Negative	Positive	Negative	Positive	Negative	Positive	Negative
Positive	23	25	48	0	47	1	47	1
Negative	0	71	35	36	35	36	35	36
Sensitivity	47.91%		100%		97.91%		97.91%	
Specificity	100%		50.70%		50.70%		50.70%	
PPV	100%		57.83%		57.31%		57.31%	
NPV	74%		100%		97.29%		97.29%	

However, the sensitivity and specificity of smear microscopy, culture, CBNAAT, and LPA when compared to real-time multiplex PCR were 27.7%/100%, 57.8%/100 %, 98.8%/100%, and 98.8%/100%, respectively (Table 2).

**Table 2:** Comparison of sensitivity, specificity, PPV and NPV of smear microscopy, culture, CBNAAT and LPA while real-time multiplex PCR considered to be a gold standard PCR - polymerase chain reaction; CBNAAT - cartridge based nucleic acid amplification test; LPA - line probe assay; NTM - nontuberculous mycobacteria; PPV - positive predictive value; NPV - negative predictive value

Real-time multiplex PCR, n=119	Smear microscopy, n=119		Culture, n=119		CBNAAT, n=119		LPA, n=119	
	Positive	Negative	Positive	Negative	Positive	Negative	Positive	Negative
Positive	23	60	48	35	82	1	82	1
Negative	0	36	0	36	0	36	0	36
Sensitivity	27.7%		57.83%		98.79%		98.79%	
Specificity	100%		100%		100%		100%	
PPV	100%		100%		100%		100%	
NPV	37.5%		50.70%		97.29%		97.29%	

**Figure 2:**
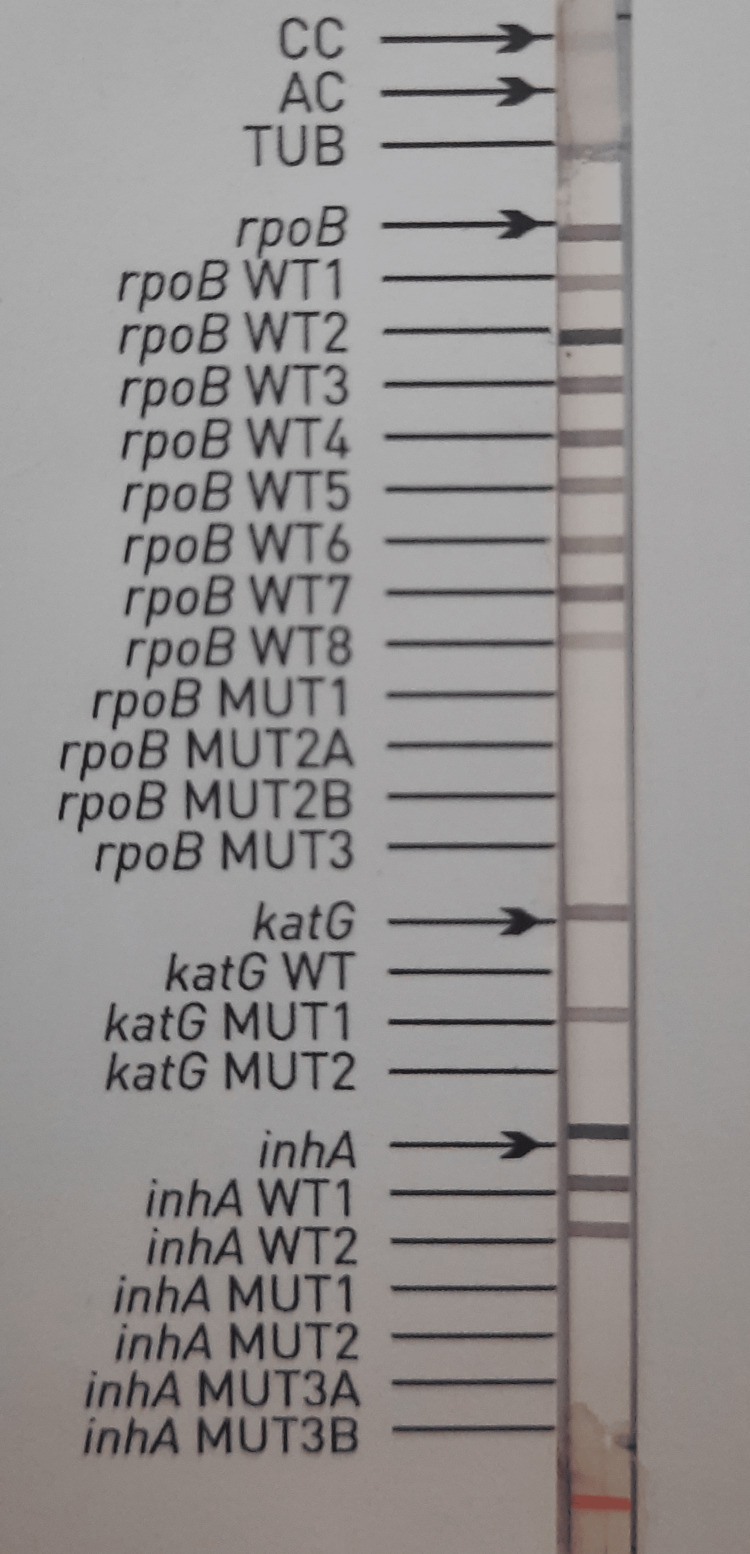
Rifampicin-sensitive INH-resistant isolate showing the presence of katG MUT1 band and absence of katG WT band, indicating mutation at the S315T1 codon of the katG gene CC - conjugate control; AC - amplification control; TUB - m. tuberculosis complex; WT - wild-type probe; MUT - mutation probe; katG - catalase-peroxidase; S315T1 - amino acid change at codon 315 from serine to threonine; rpoB - β-subunit of DNA-dependent RNA polymerase; inhA - NADH-dependent enoyl-acyl-carrier-protein reductase

Further, on combined LPA and CBNAAT, out of 82 samples, 45 (55.0%) isolates were completely susceptible to first-line ATT. One isolate was rifampicin (RIF) sensitive on CBNAAT but MDR on LPA. Hence, 46 isolates were sensitive on CBNAAT, whereas the number decreased to 45 on LPA. Interestingly, among the rest of the 37 isolates, eight isolates were RIF mono-resistant (MDR), and 22 were resistant to both rifampicin and INH (MDR), whereas seven isolates were INH mono-resistant. Hence, the MDR percentage among the MTB-positive isolates in our cases was 36.1% (30/83). Alarming was that one of the RIF-resistant isolates had the presence of strong wild-type (WT) 3 and WT4 bands; however, the corresponding mutation band MUT1 was also present. This isolate was RIF-sensitive on CBNAAT (Figure 2).

The mutations profile by resistant isolates on LPA were examined, and it was noticed that all the RIF-mono-resistant isolates had a mutation at the S531L codon of the β-subunit of DNA-dependent RNA polymerase (rpoB) gene, while one RIF-mono-resistant isolate had a mutation at the amino acid change at codon 516 from aspartic acid to valine (D516V) codon of the rpoB gene, whereas all INH-mono-resistant isolates had a mutation at the amino acid change at codon 315 from serine to threonine (S315T1) codon of the catalase-peroxidase (katG) gene. All MDR-TB strains (22/45) carried a mutation at the S531L codon of the rpoB gene and S315T1 codon of the katG gene except one MDR-TB isolate had D516V codon mutation, but also the presence of rpoB WT3/WT4 band.

## Discussion

We explored an algorithm using clinical, radiological, and molecular-based methods, viz. real-time multiplex PCR, CBNAAT, and LPA, for early diagnosis of MSTB in our study. Among clinically suspected MSTB patients, 70% had findings suggestive of an infectious pathology in imaging studies. The findings mainly comprised joint effusion, soft tissue swelling, bony erosions, vertebral collapse, synovitis, and paravertebral abscess. Our findings were in line with other studies in which similar findings suggested infectious pathology reported on imaging of EPTB suspects [9,10].

Real-time multiplex PCR yielded maximum positivity of 70% from samples of clinical suspicion and positive radiological findings of infectious pathology of the musculoskeletal region. All samples of patients with radiological findings suggestive of TB amplified MTB on all molecular modalities. On comparing real-time multiplex PCR with culture, the gold standard for diagnosis of MTB, sensitivity was better than smear microscopy (100% and 48%, respectively). Smear microscopy, although a cost-effective and easily accessible tool, has a major disadvantage of being low in sensitivity, especially in EPTB samples [11].

Similar findings were observed for CBNAAT with a sensitivity of 98% but low specificity (50.7%) than when compared with culture. The reason is that culture needs a higher bacillary load for detection, especially in EPTB samples, whereas molecular methods, viz. real-time multiplex PCR or commercially available nucleic acid amplification test, have a lower limit of detection. This has been the findings in other studies, too [12]. 

However, the specificity of smear microscopy was better than real-time multiplex PCR when compared with a gold standard (100% and 50.7%, respectively). Similar findings were observed for CBNAAT and LPA, too, with a specificity of 50.7% for both. The reason for the low specificity of real-time multiplex PCR in our study is a low positivity rate of 40.33% of culture among the MSTB suspects. Calculated sensitivity and specificity of real-time multiplex PCR employing culture as the "gold standard" were 100% and 50.7%. Various studies highlighting the role of PCR-based methods in the diagnosis of EPTB samples have been published, but the role of this modality in radiologically suspected subjects in clinching the diagnosis of MSTB still remains unexplored. In 2013, WHO endorsed GeneXpert for EPTB diagnosis leading to an era of published studies exploring its role in MSTB [13].

Our study was notable as we compared the three PCR-based methods, viz. real-time multiplex PCR, CBNAAT, and LPA, and found that the cumulative diagnostic yield of the three methods was high as compared to smear microscopy and culture in radiologically suspected MSTB. Individually each modality viz. real-time multiplex PCR, CBNAAT, and LPA fetched a diagnostic yield of 69.74%, 68.90%, and 68.90%, respectively, and were 100% concordant with each other except for one sample, which was negative on CBNAAT and LPA but amplified NTM on real-time multiplex PCR. MRI of this patient revealed findings of signal abnormalities of the second and third lumbar vertebral bodies with abscess.

Although NTM has been reported as an important pathogen in PTB samples, musculoskeletal infections with NTM are rarely reported, but our laboratory is sensitized to NTM diagnosis in EPTB samples, and a similar finding has been reported in EPTB samples in one of our previous studies, too [4,14,15]. Thus, it is imperative that the possibility of NTM in MSTB samples needs to be explored and all possible measures taken to identify this pathogen at all laboratories.

Further, the DST analysis was obtained by the cartridge based method and compared with genotypic susceptibility analysis by LPA. Although there is still a dearth of DST studies in MSTB samples, one of the authors, Mohan et al. in his study reported 78.3% of MDR isolates and 2.7% of extensively drug-resistant XDR isolates among TB spine cases [16]. The rate of drug resistance among EPTB isolates was much higher in this study. The reason might be the large sample size in their study. However, our rigid exclusion criteria in which we completely excluded samples with the possibility of secondary drug resistance might have led to lower MDR (36.1%) isolates being detected.

A notable finding of our study was that one isolate reported as RIF-sensitive on cartridge-based method was MDR on LPA. This was alarming as with the advent of the cartridge-based method, which has a shorter turnaround time, the genotypic analysis of resistant strains has taken a back stand.

With the diagnostic difficulties associated with MSTB, a process of combining imaging, preferably MRI, and the three molecular modalities, namely real-time multiplex PCR, CBNAAT, and LPA, would be a crucial diagnostic algorithm in the management of this form of TB.

Individually clinical, radiological, and molecular tools have their own limitations. A combination of these would be promising in the eradication of PTB as well as EPTB. Further, with rising drug resistance, LPA would prove to be a valuable tool as part of the algorithm for MSTB patients. Also, the role of NTM in MSTB needs to be further explored, and clinicians need to be conscious of the possibility of NTM in MSTB samples before initiating therapy.

Although our study is the first study of North India in which drug resistance in MSTB samples has been explored, it had certain limitations. It was a purely hospital-based study, and the findings can not be extrapolated to the community at large, for which a community-based study needs to be planned. Also, we could not analyze our isolates for mutations other than those offered by LPA viz. rpoB, katG, and inhA, for which targeted genomic sequencing was required, which could not be planned due to lack of facility at our center.

## Conclusions

A combination of clinical, radiological, and Molecular diagnoses is the need for an hour for MSTB diagnosis. Both NAAT and LPA are important tools for the detection of TB with high sensitivity and minimal turnaround period, along with the detection of drug-resistant TB. Although limitations like cost constraints exist in following this algorithm but bringing down the morbidity, mortality, and cost of treatment associated with MSTB, the approach seems to be sound and promising. Hence these modalities should be included in the battery of tests for early identification and DST profile of *Mycobacterium tuberculosis* in MSTB disease.
